# Effects of sensory deprivation on glomerular interneurons in the mouse olfactory bulb: differences in mortality and phenotypic adjustment of dopaminergic neurons

**DOI:** 10.3389/fncel.2023.1170170

**Published:** 2023-06-12

**Authors:** Alexandra Angelova, Marie-Catherine Tiveron, Mathieu D. Loizeau, Harold Cremer, Jean-Claude Platel

**Affiliations:** Aix-Marseille University, Centre National pour la Récherche Scientifique (CNRS), Institut de Biologie du Développement de Marseille (IBDM), Unité Mixte de Récherche 7288 (UMR 7288), Turing Center for Living Systems, Marseille, France

**Keywords:** adult neurogenesis, *in vivo* imaging, sensory deprivation, olfaction, neuronal cell death

## Abstract

Neurogenesis persists in the mammalian subventricular zone after birth, producing various populations of olfactory bulb (OB) interneurons, including GABAergic and mixed dopaminergic/GABAergic (DA) neurons for the glomerular layer. While olfactory sensory activity is a major factor controlling the integration of new neurons, its impact on specific subtypes is not well understood. In this study we used genetic labeling of defined neuron subsets, in combination with reversible unilateral sensory deprivation and longitudinal *in vivo* imaging, to examine the behavior of postnatally born glomerular neurons. We find that a small fraction of GABAergic and of DA neurons die after 4 weeks of sensory deprivation while surviving DA-neurons exhibit a substantial decrease in tyrosine hydroxylase (TH) expression levels. Importantly, after reopening of the naris, cell death is arrested and TH levels go back to normal levels, indicating a specific adaptation to the level of sensory activity. We conclude that sensory deprivation induces adjustments in the population of glomerular neurons, involving both, cell death and adaptation of neurotransmitter use in specific neuron types. Our study highlights the dynamic nature of glomerular neurons in response to sensory deprivation and provide valuable insights into the plasticity and adaptability of the olfactory system.

## Introduction

Neurogenesis persists in the mammalian brain after birth and newly generated neurons are permanently added in the different layers of the mouse olfactory bulb (Platel et al., 2019; Alvarez-Buylla and Garcia-Verdugo, 2002). Olfactory sensory activity is one of the main factors controling the integration of these newborn neurons. Indeed, pioneering work showed that olfactory sensory deprivation via unilateral naris occlusion (UNO) led to a strong reduction in size of all OB layers accompanied by massive cell loss (Meisami and Mousavi, 1981; Brunjes, 1985; Cummings and Brunjes, 1997). Newborn neurons are particularly affected by UNO with increased cell death as well as decreased dendritic length and spine density (Saghatelyan et al., 2005; Kelsch et al., 2009). BrdU pulse chase experiments after UNO also revealed a decrease in the density of newborn peri-glomerular neurons (PGN) (Bastien-Dionne et al., 2010).

Newborn PGN represent a heterogeneous population of GABAergic interneurons, that can be distinguished by the expression of subtype specific markers like calretinin and calbindin (Angelova et al., 2018). Interestingly, a major subpopulation of the GABAergic PGN expresses tyrosine hydroxylase (TH), as well as other enzymes of the catecholamine synthesis pathway, and use dopamine as second neurotransmitter (Baker, 1990).

While the general impact of UNO on the OB is well described, the consequences on chemospecific populations is far less clear. The first study to address this question showed that UNO leads to an important loss of TH expression, but only partially of dopamine decarboxylase, another enzyme involved in dopamine synthesis, suggesting an absence of death in the TH population (Baker, 1990). Nevertheless, more recent studies reported a reduction in numbers of newly generated TH-positive PGN but not calretinin or calbindin-expressing PGN (Bovetti et al., 2009; Bastien-Dionne et al., 2010) suggesting that only TH-positive newborn PGN depend on olfactory input for their survival. However, since TH protein expression is dependant on olfactory input, conventional TH-immunostaining did not allow for accurate assessment of the number of DA neurons after naris occlusion (Baker, 1990). To circumvent this problem, new genetic strategies have been applied. By labeling dopaminergic neurons using DAT-tdTomato, it was shown that brief sensory deprivation led to a minor drop in immunofluorescence for the dopamine-synthesizing enzyme dopamine decarboxylase (DDC) and a sustained decrease for TH (Byrne et al., 2022). Using TH-Cre mice to induce expression of a fluorescent reporter, Sawada and colleagues showed with an histological approach that 40% of DA neurons disappear upon UNO (Sawada et al., 2011). Taken together, there is a lack of consensus concerning the effect of sensory deprivation on different PGN populations, and specifically on DA neurons.

The combination of *in vivo* two-photon imaging and genetic labeling techniques has provided a powerful means to address the impact of sensory deprivation on specific neuronal populations. In a previous study (Angelova et al., 2019), we used this approach to elucidate the differential effects of sensory deprivation on glomerular neurons. Our findings revealed that glutamatergic neurons positive for NeuroD6 were resistant to cell death during sensory deprivation, whereas GABAergic neurons were susceptible. Building upon these previous results, the present study focused on the behavior of dopaminergic (DA) neurons and other GABAergic neurons throughout the course of reversible sensory deprivation. We discovered that sensory deprivation induced the death of 15% of GABAergic neurons and only 5% of DA neurons. Furthermore, we observed a strong decrease in TH expression, which serves as an indicator of dopaminergic activity. Crucially, when the nasal occlusion was reversed, we observed an arrest of cell death across the neuronal population and a restoration of TH-GFP expression levels to their initial state in the surviving neurons.

## Results

**FIGURE 1:**
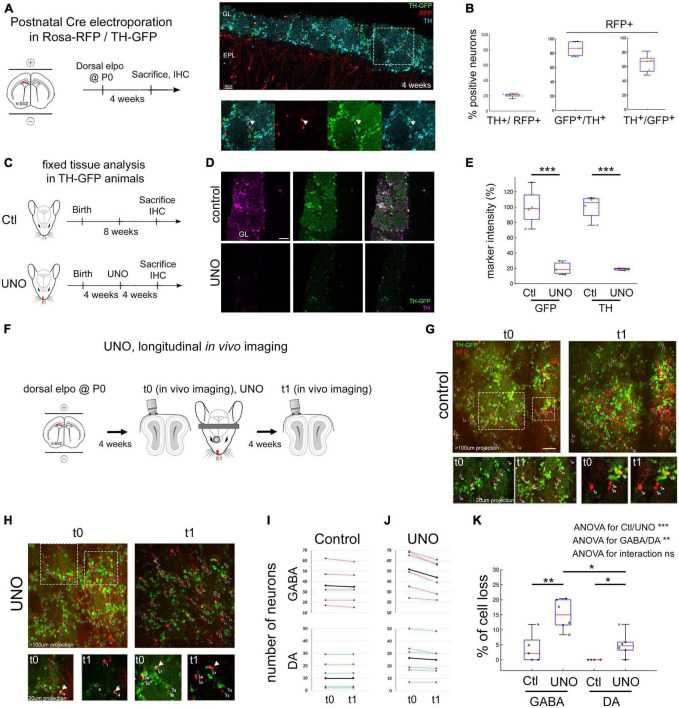
Unilateral naris occlusion reduces immuno-TH and TH-GFP intensity and trigger GABA and DA neurons death. **(A)** Dorsal electroporation (elpo) of Cre plasmid into postnatal Rosa-RFP/TH-GFP mice is performed at P0 and animals are sacrificed at 4 weeks. **(B)** Four weeks after elpo, RFP+ and RFP+ GFP+ interneurons are observed in the glomerular layer of the OB. TH expression of these neurons can be confirmed with anti-TH immunostaining. **(C)** Quantification of TH, TH-GFP and RFP labeling on fixed tissue. Around 20% of RFP+ neurons are positive for immuno-TH. 86% of RFP+ TH+ neurons are GFP+ and 64% of RFP+ GFP+ neurons are TH+. Each dot is the mean measurement of one animal. **(D)** Experimental timeline in Ctl/UNO experiments. **(E,F)** TH-GFP and immuno-TH are reduced to 20% of original intensity levels after 4 weeks of UNO. **(G)** Experimental approach to monitor cell survival *in vivo* after UNO. **(H,I)** *In vivo* observation of OB interneurons in control animals and before (t0) and after UNO (t1). Circles represent neurons that disappear. Arrowheads in I highlight neurons with a decrease of GFP expression. **(J)** Individual animals and the amount of neurons observed in the course of 4 weeks in control condition and under UNO. Black line represents the mean number of neurons. Neurons were classified as GABA (RFP+ only) and DA-neurons (RFP+ GFP+). **(K)** Quantification of mean number of cell loss in GABA and DA-neurons under control and UNO condition. GABA and DA-neurons are lost after UNO although significantly less DA-neurons than GABA neurons are lost. Statistics: 2 way ANOVA on data ranks followed by Bonferroni *post-hoc* tests: *p < 0.05. **p < 0.01. ***p < 0.001. In panels **(B,E,K)** each dot is the mean measurement of one animal. Scale bars: 50 μm **(B,E)**; 40 μm **(I)**. IHC: Immuno-histo-chemistry.

The dorsal region of the lateral ventricle generates predominantly dopaminergic and non-dopaminergic GABAergic glomerular neurons during postnatal development. To label this progenitor population, we performed postnatal electroporation of a Cre expression plasmid into the dorsal ventricular wall of Rosa-RFP/TH-GFP double transgenic mice (Figure 1A). Four weeks later, 20.6% ± 1.2 (mean ± sem) of RFP positive neurons were immunopositive for TH after migration in the glomerular layer (GL). Of these RFP+/TH+ neurons, 86.2% ± 4.5 were also positive for GFP, demonstrating that TH-GFP is a reliable marker for endogenous TH expression in OB interneurons (Figures 1B, C). We also observed that 36% ± 5.8 of TH-GFP neurons lacked TH immunostaining, supporting the idea that TH-GFP expression in DA neurons precedes the appearance of TH protein (Baker et al., 2001; Figure 1C right). Calretinin or calbindin were not expressed in any of the TH-GFP positive cells (data not shown), confirming previous findings in a different TH-GFP mouse line (Sawada et al., 2011). Consistent with previous studies (Tiveron et al., 2017), we considered the remaining RFP+ PGN as purely GABAergic neurons. Thus, the electroporation approach allowed us to distinguish DA neurons from GABAergic neurons in the OB glomerular layer. To investigate the effect of olfactory sensory deprivation on TH protein and TH-GFP levels in the GL, we performed unilateral naris occlusion (UNO) by inserting a polyethylene plug into the naris of TH-GFP mice four weeks after Cre-electroporation at P0 (Cummings and Brunjes, 1997; Figure 1D). Another 4 weeks after UNO, TH-immunofluorescence intensity in the GL was significantly reduced to 18.8% ± 0.8 of controls (Figures 1E, F; *p* < 0.001). This decrease in TH-protein was paralleled by an 80% ± 5 reduction in TH-GFP levels (Figure 1F, *p* < 0.001), validating that TH-GFP accurately reflects protein expression and confirming previous findings (Saghatelyan et al., 2005).

To investigate the impact of sensory deprivation on the survival of individually identified DA and GABAergic neurons in the GL, we used longitudinal *in vivo* 2-photon imaging of postnatally electroporated Rosa-RFP/TH-GFP mice. We acquired Z-stack volumes of large field of views (600 μm × 600 μm), covering the entire depth of the GL, immediately before (t0, 4 weeks post-electroporation) and 4 weeks after UNO (t1, 8 weeks post-electroporation; Figure 1G). Specific glomeruli were identifiable based on the circular organization of GFP expressing neurons. Individual RFP+ neurons were reliably identified over time, based on their morphology and relative position to neighboring cells (Figures 1H, I). As observed previously, we noted a slight expansion in the size of glomeruli, an increase in distance between labeled neurons in control condition (Platel et al., 2019) but a marked shrinkage under UNO (Angelova et al., 2019). We quantified the percentage of cell loss under control and UNO condition and between cell types, GABA versus DA. We performed a 2-way ANOVA on data ranks with ctl/UNO and cell-type as fixed effects. This revealed a significant effect of both “ctl/UNO” (*p* < 0.001) and “cell type” (*p* < 0.01), showing that “UNO” increased cell death and that cell death is higher in GABAergic neurons. However, the interaction between the two fixed effects was not significant, suggesting that deprivation does not trigger a population-specific cell death response. In control condition, observation of 229 RFP+ GABAergic neurons, including 49 DA-neurons (RFP+ GFP+) in 5 mice demonstrated that cell loss was very low, with 3.75% ± 2.2 of the GABAergic neurons (4/180, RFP+ GFP−) and no loss of DA neurons (0/49, RFP+ GFP+, Figures 1H, J). GFP intensity in control animals was stable over time (similar GFP intensity at t0 and t1 in Figure 1H). Four weeks after UNO, we quantified that 15% ± 2.2 of RFP+ GABAergic neurons were lost (47/310, 6 animals, *p* < 0.01 compared to Ctl GABA, Figures 1I–K). DA neurons showed only a moderate loss, about 5% ± 1.7 (*n* = 9/158 neurons in 6 animals, Figures 1I–K, *p* < 0.05 compared to ctl DA and, *p* < 0.05 compared to UNO GABA). The striking reduction in TH-GFP expression that was detected immunohistologically was also obvious by direct *in vivo* observations (Figure 1I arrowheads).

**FIGURE 2:**
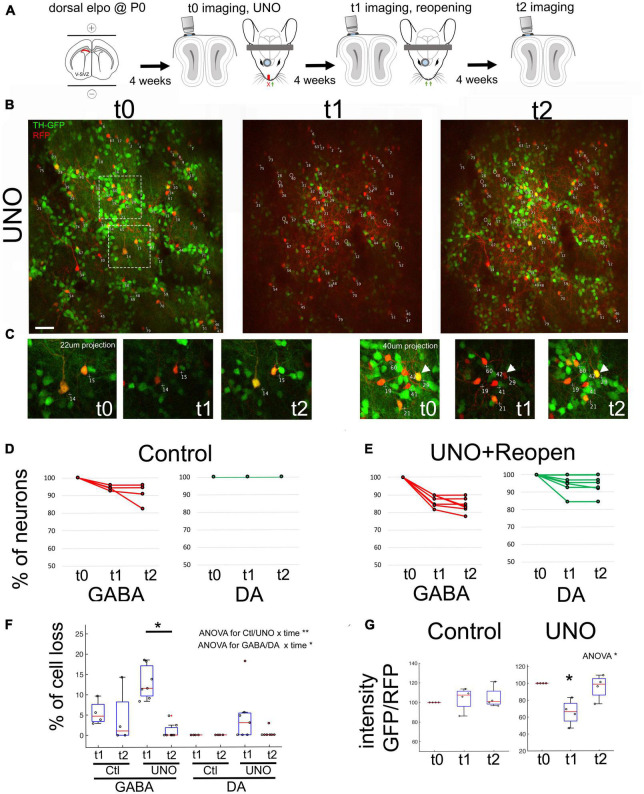
Recovery of sensory activity leads to re-expression of TH in the same neurons. **(A)** Experimental approach depicting UNO and reopening. **(B)** *In vivo* observation of the same neurons before UNO (t0), after UNO (t1) and after reopening of naris (t2). Note the strong reduction of GFP in t1 and its re-appearance in t2. **(C)** Close up of surrounded region in **(B)**. Arrows shows examples of DA neurons exhibiting TH expression variation after UNO and after reopening. **(D,E)** Quantification of observed GABA and DA neurons across t0-t1-t2 in control as well as after UNO and reopening within individual animals. **(F)** Percentage of cell loss in GABA and DA neurons across t0-t1-t2 in control and after UNO and reopening. Cell loss in the GABAergic population is significantly reduced after reopening. Statistics: 2 way ANOVA on data ranks with repeated measures followed by a Wilcoxon rank sum test corrected for multiple comparisons. **(G)** Ratio of GFP/RFP intensity across t0, t1 and t2. Note the strong reduction of GFP in t1 and its re-appearance in t2. Statistics: ANOVA on data ranks with repeated measures followed by a Wilcoxon rank sum test corrected for multiple comparisons. *p < 0,05; **p < 0,01. Scale bar = 20 μm.

To further explore the plasticity of the glomerular neuronal network, we decided to perform reversible UNO (Figure 2A). This would enable us to investigate the impact of sensory activity recovery on cell survival and on TH-GFP expression. We performed the same *in vivo* imaging approach as described above (t0, imaging and UNO), but removed the nasal plug after imaging at t1 (Figure 2A). The same field of view was then imaged four weeks after reopening (t2, *n* = 7, Figures 2B, C). Control Rosa-RFP/TH-GFP animals (*n* = 6) were submitted to the same labeling and imaging protocol in the absence of UNO. First, we analyzed the presence of GABAergic and DA-neurons over the post-opening period t1-t2. We performed a 2-way ANOVA on data ranks with repeated measures (time) analysis with ctl/UNO and cell-type as fixed effects. We found a significant effect between “ctl/UNO × time” interaction (*p* < 0,01) and “cell type × time” interaction (*p* < 0,05). Under control conditions, quantification of RFP cell numbers revealed that overall cell loss at t2 was detected at low levels in all observed animals (GABAergic: 1%, DA: 0.0%, Figures 2D, F). Four weeks after reversal of UNO, cell loss was almost non-detectable in both populations (GABAergic: from 13% ± 1.7 at t1 to 1% ± 0.8 at t2, *p* < 0,05, DA: from 4.5 ± 2.6 to 0.4 ± 0.4%, Figures 2E, F). Thus, the impact of UNO on neuronal death was abruptly reversed when sensory information was reinstalled.

To assess the evolution of TH expression, we calculated the intensity ratio of GFP over RFP in individual DA-neurons. We performed an ANOVA on data ranks with repeated measures (t1-t2-t3) analysis with ctl/UNO as fixed effects and found an interaction “ctl/UNO × time” (*p* < 0,05). In control animals, the GFP/RFP ratio was unchanged at all three observation time points, as expected (Figures 2E, F; control: *n* = 58 cells, 4 animals UNO: *n* = 57 cells, 4 animals). After UNO, we observed a significant 33% ± 7 relative loss in GFP intensity in cell bodies (*p* < 0.05, Figures 2B, G). Four weeks after reopening (t2), GFP fluorescence in the identified RFP+ GFP+ neurons returned to pre-UNO levels (95.6 ± 7.2% of controls, Figure 2C right arrow and Figure 2G). Thus, the reduction in TH expression observed in the OB after UNO indicates that there is a specific and transitory adjustment in the use of this neurotransmitter in this neuronal population.

## Discussion

In this study, we combined sensory activity manipulation, genetic labeling and long-term *in vivo* observations, to follow the fate of individually identified GABAergic and DA PGN. Using this approach we demonstrate for the first time that dopaminergic PGN dynamically modulate their level of TH expression as a function of sensory input and present a low level of cell death, as observed in GABAergic neurons.

All studies addressing the effect of UNO unambiguously show a decrease in TH expression. We demonstrate that this decrease in TH expression can also be observed *in vivo*, providing a tool for monitoring the dynamic expression of TH over prolonged time windows.

The issue of the level of death of DA-neurons after UNO represents a central theme in the relevant literature. Here, we demonstrate that few DA neurons die after UNO, supporting early studies by the Baker laboratory (Baker, 1990) who elegantly showed that aromatic L-amino acid decarboxylase, the second enzyme involved in the dopamine biosynthetic pathway, was still expressed after deprivation.

Several previous studies based on histological approaches used TH expression to detect the survival or death of DA neurons after UNO (Bovetti et al., 2009; Bastien-Dionne et al., 2010). However, as TH expression is modulated by sensory activity, the use of activity independent markers is needed to distinctly reflect the presence of dopaminergic neurons presence. In line with this concept, the study of Sawada and colleagues combined the use of VGAT-venus mice with *in vivo* imaging to follow the entire population of GABAergic PGN after UNO, including DA-neurons. They observed around 11% of lost cells, very similar to our own observation. Interestingly, the use of TH-Cre:R26R-CFP mice, to permanently label specifically DA-neurons, led to a different result. Indeed, histological analyses of these animals showed that 40% of the DA neurons died after UNO. However, age differences in the experimental animals, (postnatal here versus adults in the previous study) or differences in the genetic strategy might explain the contradictory findings. Moreover, recent work demonstrated that BrdU, which has been applied in most histology based analyses to determine neuronal age, is a highly toxic agent, that leads to considerable cell death when used in long term experiments (Platel et al., 2019). It is possible that some of the cell loss accredited to sensory deprivation was due to this toxicity.

Finally, the fact that the loss of TH expression is reversible after restoration of sensory activity demonstrates that DA neurons do not change their identity, but transiently shut down the TH neurotransmitter pathway.

What could the physiological relevance of this neuronal death and of the decrease of TH expression in DA-neurons? Overall the fact that cell death is more important in GABAergic neurons than in dopaminergic or glutamatergic glomerular neurons (Angelova et al., 2019) shows unequivocally that the OB does not enter into a state of general apoptosis under sensory deprivation. Instead, we can hypothesize that the OB system maintains a complex homeostatic balance between excitation and inhibition, finely tuned by sensory activity. Sudden local imbalance between excitation and inhibition, caused by sensory deprivation, could lead to an excessive increase in inhibition in the network. In this scenario, decreased environmental excitation must be balanced by an overall decrease in bulbar inhibition, allowing the system to continue to transmit information to higher order brain areas. Given that adult neurogenesis continuously supplies the OB with inhibitory interneurons, directed apoptosis of some of these cells may be a feasible mechanism to finetune inhibition in the OB system. An alternative mechanism for reducing inhibition would be a decrease in dopamine synthesis as TH controls the rate limiting steps for its production.

Taken together, we demonstrate that the glomerular neuronal network is capable of a strong reaction to alterations in sensory activity. These adaptations involve plasticity changes in the dopaminergic neurotransmitter phenotype and loss of inhibitory neurons from the network, which could allow to maintain the excitability of the system necessary for efficient signal transmission.

## Materials and methods

### Animals

All mice were treated according to protocols approved by the French Ethical Committee (#5223–2016042717181477 v2). Mice were group-housed in regular cages under standard conditions, with up to five mice per cage on a 12 hr light–dark cycle. Rosa-red fluorescent protein (RFP) mice [Ai14, Rosa26-CAG-tdTomato (Madisen et al., 2010)] and TH-green fluorescent protein (GFP) transgenic mice (3846686) were obtained from the Jackson Laboratory and used on a mixed C57Bl6/CD1 background. All experiments were performed on males and females.

### Postnatal electroporation

*In vivo* electroporation was performed as previously described (Boutin et al., 2008). Briefly, P0-P1 pups were anesthetized by hypothermia and 2 μl of pCAG-CRE plasmid (#13775 Addgene, at 4 μg/μl) were injected in the lateral ventricle. Animals were subjected to five 95V electrical pulses (50 ms, separated by 950 ms intervals) using the CUY21 edit device (Nepagene, Chiba, Japan) and 10 mm tweezer electrodes (CUY650P10, Nepagene) coated with conductive gel (Control Graphique Medical, France). Electrical pulses were applied to target the dorsal V-SVZ. Electroporated animals were then reanimated in a 37°C incubator before returning to the mother.

### Cranial window implantation

Implantation of a cranial window was performed as previously described (Drew et al., 2010) with minor modifications. Briefly, 4 week old mice were anesthetized by intraperitoneal (ip.) injection of ketamine/xylazine (125/12.5 mg/kg). Dexaméthasone (0.2 mg/kg) and buprenorphine (0.3 mg/mL) were injected subcutaneously and lidocaine was applied locally onto the skull. The pinch withdrawal reflex was monitored throughout the surgery, and additional anesthesia was applied if needed. Carprofen (5 mg/kg) was injected ip. after the surgery. A steel head-fixation bar was added attached to the skull with dental cement (Superbond, GACD). A 2.5 mm × 1.5 mm piece of skull overlying the OB was carefully removed using a sterile scalpel blade. Great care was taken not to damage the dura. A thin layer of low toxicity silicon adhesive (Kwik-Sil) was applied over the craniotomy and covered with a round 3 mm coverslip. The craniotomy was sealed with superglue and dental cement (Superbond, GACD). The first time point (t0) of our microscopic observation was performed after surgery on these anesthetized mice. UNO was performed following cranial window surgery.

### Unilateral naris occlusion

For olfactory sensory deprivation, a polyethylene tube was inserted (BD Intramedic, PE50, 3 mm long) into one naris and sealed with cyanoacrylate glue (Cummings et al., 1997). Efficiency of occlusion was checked the following day and before each imaging session by the absence of air bubbles after application of a water drop on the occluded naris. Occlusion lasted for 4 weeks. At the end of the experiment immunostaining against tyrosine hydroxylase was performed to further confirm the efficiency of the occlusion. For reversible occlusions, mice were anesthetized after 4 weeks of occlusion and the polyethylene tube was carefully removed. If the quality of the cranial window has declined due to regrowth of connective tissue and bone, a new surgery was performed to reopen the window and allow for subsequent imaging.

### *In vivo* two-photon imaging

We used a Zeiss LSM 7MP two-photon microscope modified to allow animal positioning under a 20× water immersion objective (1.0 NA, 1.7 mm wd) and coupled to a femtosecond pulsed infrared tunable laser (Mai-Tai, SpectraPhysics). After two-photon excitation, epifluorescence signals were collected and separated by dichroic mirrors and filters on 4 independent non-descanned detectors (NDD). Images were acquired using an excitation wavelength of 950 nm. GFP was collected at 500–550 nm. RFP was first collected between 605–678 nm. In addition, we collected an additional RFP signal between 560–590 nm that was voluntarily saturated to allow a better identification of subcellular structures like dendrites. In general, image acquisition lasted about 10 min. The imaging window was centered on the dorsal surface of the OB. The whole peri-glomerular layer was imaged (around 150 μm depth).

For longitudinal observation, we used the same principle as applied in Platel et al. (2019). In short, the same field of view was localized based on the geometric motifs of groups of neurons and specific morphological features of individual cells. Images of 606 μm × 606 μm were acquired at 0.59 μm/pixel resolution in the xy dimension and 2 μm/frame in the z dimension to a maximal depth of 200 μm.

### Chronic *in vivo* imaging analysis

Quantitative analyses were performed on raw image stacks using FIJI software (Schindelin et al., 2012). All neurons identified on the first image were assigned a number using ImageJ overlay. Based on morphology and relative position each neuron was individually numbered and tracked during the successive imaging sessions. They were then labeled as TH-GFP positive or not. Detection of lost cells was performed solely on RFP expression. Results were summarized in Microsoft Excel. Occasionally neurons located at the border of an image were placed outside of the imaged field in one of the following sessions. These cells were excluded from further analyses. Animals showing an evident degradation of the imaging window were excluded from further imaging sessions.

### GFP fluorescence evolution

To determine the effect of UNO on GFP intensity and to be independent of laser power, we used one wavelength to excite both GFP and RFP and performed ratio calculation as follow: we drew region of interest over the cell bodies of GFP positive neurons. We extracted both the fluorescence intensity of the red channel and of the green channel at t0, t1 and t2. We then performed a ratio green/red for each time point for each cell. We then normalized the ratio for each cell to t0 ratio. Finally we averaged this values per animal (4 control animals and 4 UNO animals).

### Immunohistochemistry and image analysis

For histological analysis, mice were deeply anesthetized with an xylazine/ketamine overdose. Intracardiac perfusion was performed with 4% paraformaldehyde in PBS. The brain was collected and incubated overnight in the same fixative at 4°C. Sections were cut at 50 μm sections were cut with a microtome (Microm). Standard immunostaining protocols were performed on coronal free floating sections. Briefly, sections were rinsed in PBS and incubated in a blocking buffer [10% fetal bovine serum (FBS), 0.3% Triton X-100 in PBS] for 1 h. Subsequently, sections were incubated in primary antibody solution [5% FBS, 0.1% Triton X-100 in PBS (PBST)] and Tyrosine Hydroxylase antibody (chicken Ig, AVES; 1:1,000) overnight at 4°C. The following day, sections were rinsed 3 times in PBS and incubated with species-appropriate secondary antibody in PBST for 2 h at RT using gentle rocking. Alexa fluor-conjugated secondary antibodies were purchased from Life Technologies. Nuclear counterstain HOECHST 33258 (Invitrogen, 1:2,000) was added before sections were washed in PBS and mounted using Mowiol as a mounting medium. Optical images were taken either using a fluorescence microscope (Axioplan2, ApoTome system, Zeiss) or a laser confocal scanning microscope (LSM510 or LSM780, Zeiss, Germany) using same settings between the conditions. Image analysis was performed using FIJI software (Schindelin et al., 2012). All experiments and quantifications were performed blindly to experimental groups. For Figures 1E, F, immunofluorescence were performed on the same day with the same antibody dilution. Regions of interest were drawn over the whole glomerular layer and the mean intensity of GFP or TH compared between control and UNO animals (4 control and 3 UNO animals).

### Statistical analyses

All measurements are presented as mean ± standard error of the mean. In box plot representation, center red line represents the median; box limits represents upper and lower quartiles and whiskers, outliers. Statistical comparisons were performed using Matlab. In Figure 1F, we used a Wilcoxon rank sum test. In Figure 1K we used 2-way ANOVA on data ranks with UNO and cell-type as fixed effects followed by Bonferroni *post-hoc* tests. In Figure 2E, we used 2-way ANOVA on data ranks with repeated measures (t1-t2) and with UNO and cell-type as fixed effects followed by Wilcoxon rank sum test corrected for multiple comparison. In Figure 2F, we used ANOVA on data ranks with repeated measures (t1-t2-t3) followed by Wilcoxon signed rank test corrected for multiple comparison.

## Data availability statement

The raw data supporting the conclusions of this article will be made available by the authors, without undue reservation.

## Ethics statement

The animal study was reviewed and approved by the Comité d’éthique en expérimentation animale de Marseille n°14.

## Author contributions

AA, M-CT, ML, and J-CP performed experiments. AA, M-CT, and J-CP analyzed the data. J-CP, AA, and HC conceived the project. J-CP and HC wrote the manuscript. All authors commented on the manuscript and approved the submitted version.
